# Fiber Optic-Based Refractive Index Sensing at INESC Porto

**DOI:** 10.3390/s120608371

**Published:** 2012-06-18

**Authors:** Pedro A. S. Jorge, Susana O. Silva, Carlos Gouveia, Paula Tafulo, Luis Coelho, Paulo Caldas, Diana Viegas, Gaspar Rego, José M. Baptista, José L. Santos, Orlando Frazão

**Affiliations:** 1 INESC Porto, Rua do Campo Alegre 687, Porto 4169-007, Portugal; E-Mails: ssilva@fc.up.pt (S.O.S.); icarlos07@gmail.com (C.G.); paula.tafulo@fc.up.pt (P.T.); lccoelho@gmail.com (L.C.); pcaldas@inescporto.pt (P.C.); diana.viegas@gmail.com (D.V.); gaspar@estg.ipvc.pt (G.R.); jmb@inescporto.pt (J.M.B.); josantos@fc.up.pt (J.L.S.); ofrazao@inescporto.pt (O.F.); 2 Faculdade de Ciências da Universidade do Porto, Rua do Campo Alegre 687, Porto 4169-007, Portugal; 3 Centro de Competência de Ciências Exatas e da Engenharia, Universidade da Madeira, Campus da Penteada, Funchal 9000-390, Portugal; 4 Escola Superior de Tecnologia e Gestão de Viana do Castelo, Av. do Atlântico, Apartado 574, Viana do Castelo 4900-348, Portugal

**Keywords:** refractive index measurement, Bragg gratings, long period gratings, surface plasmonic resonance, multimode interference, fiber Fabry-Perot interferometers

## Abstract

A review of refractive index measurement based on different types of optical fiber sensor configurations and techniques is presented. It addresses the main developments in the area, with particular focus on results obtained at INESC Porto, Portugal. The optical fiber sensing structures studied include those based on Bragg and long period gratings, on micro-interferometers, on plasmonic effects in fibers and on multimode interference in a large spectrum of standard and microstructured optical fibers.

## 1. Introduction

In 1983, Cooper *et al.* [1] proposed the first optical fiber refractometer to measure the refractive index (RI) in several liquids. This configuration is very complex and combines two technologies (bulk and fiber). In the following year, Kumar *et al.* [2] described the first all-fiber refractometer using a single biconical filter fabricated in a multimode fiber. During the last three decades several researchers have published different types of all-fiber sensors for refractive index measurement. The technology includes a variety of configurations such as fiber taper structures [2]; Bragg grating (FBG) structures [3]; long period gratings (LPG) in a simple fiber structure or combined in series with another LPG forming a Mach-Zehnder interferometer [4]; multimodal interference using a simple structure of single mode-multimode-single mode fibers spliced in sequence (SMS); micro-interferometers based on chemical etching [5] or on microstructured fibers [6]; and including also the exploitation of surface plasmon resonance (SPR) [7] effects on thin-film deposited on different fiber structures.

In this article, a review of the developments in fiber optic based refractive index sensing undergone at INESC Porto/Portugal is presented. It provides an integrated overview of fiber sensing structures targeting such measurand, with presentation of selected results published elsewhere along the years. Considering the characteristics of the different sensor configurations, distinct approaches were possible in the organization of the results. We have chosen to divide the work presented here considering some distinctive elements used in each case rather than common properties. Therefore result presentation comprises section on sensors based on: FBG, LPG; multimodal interference; fiber micro cavities; cladding modes and plasmonic structures.

## 2. Fiber Bragg Grating Structures

Fiber Bragg gratings are simple, versatile, small and intrinsic sensing elements that can be written in optical fibers and, consequently, have all the advantages normally attributed to fiber sensors. In addition to the fact that typically the measurand information is encoded in the resonant wavelength of the structure, which is an absolute parameter, these devices are inherently self-referenced. Moreover, there are several intrinsic advantages associated with FBG technology such as operation in reflection, narrowband spectral response and their compatibility with standard telecom technology, and the intrinsic multiplexing capability, particularly important in the context of remote, distributed and multi-parameter sensing [8]. Typically, FBG-based refractometers rely on the evanescent field of the core mode under fiber etching conditions, which enables interaction with the surrounding medium.

The first demonstration of an FBG as a refractometer was done in 1997 by Asseh *et al.* [3], and it was based on the application of chemical etching to the fiber region where the grating was located. After etching the fiber had a diameter of ∼11 μm, thus ∼1 μm of cladding still remained. The sensor was tested in different solutions of sucrose, inferring a variation of refractive index between 1.333–1.345. The estimated sensitivity was 1 nm/RIU (RIU—Refractive Index Unit) and the measured resolution was 5 × 10^−4^ RIU. In Portugal the first approach to this type of sensor was presented by Pereira *et al.* [9]. The sensing head contains two in-line FBGs, with one of them sensitive only to temperature, while the other is also sensitive to salinity through the associated changes in the refractive index of the water. The refractive index sensitive grating was etched and presented a highly reduced diameter (∼8 μm), allowing great interaction of the evanescent field with the surrounding liquid; the second grating was used for temperature measurements. The sensor was subjected to different concentrations of salt which implies a variation of refractive index in the range 1.330–1.341. The sensor showed sensitivity to refractive index of roughly 727 nm/RIU and a resolution of 10^−4^ RIU. Frazao *et al.* [10] demonstrated another scheme using a single FBG written in side-hole fiber for simultaneous measurement of refractive index and temperature. Etching the fiber cladding at the position of the Bragg grating enabled the evanescent field of the guided mode to interact with the immediate surrounding environment of the fiber. When the fiber grating was immersed in a liquid sample, this resulted in a wavelength response of the Bragg grating that was affected by the refractive index of the solution to be measured. In this scheme, the sensing head was based on a FBG written on an H-shaped fiber. Exposing the Hi-Bi side-hole fiber to chemical etching formed the H-shaped fiber. The two holes of the side-hole fiber are open by the etching process, allowing the evanescent field of the Hi-Bi FBG to interact with the external liquid. Owing to the birefringence of this sensing structure, each polarization mode will have different response to the measurands and it becomes possible to simultaneously measure refractive index and temperature. The sensing head was exposed to solutions with different refractive index in the range between 1.333–1.390. Sensitivities of 3.5 nm/RIU and 2.4 nm/RIU were obtained for the fast and slow axis FBG signatures, respectively, and a resolution of 5 × 10^−3^ RIU was demonstrated.

**Figure 1.**
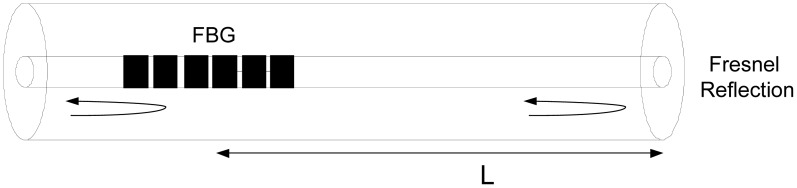
Fabry-Perot cavity based on Bragg grating and Fresnel reflection.

Recently, a different approach based on a fiber tip interaction to obtain the refractive index of the surrounding liquid by monitoring the channeled spectrum generated by an in-fiber low-finesse Fabry-Pérot FBG interferometer was studied. A temperature independent refractometer based on a low finesse Fabry-Pérot FBG cavity was proposed by Silva *et al.* [11]. In this scheme the two interfering waves are obtained from the Fresnel reflection at the distal end of a fiber probe and the reflected light from a short, low reflectivity, fiber Bragg grating written a few millimeters away from the fiber end (see Figure 1). The reflectivity of the FBG was set below 5% in order to ensure, together with the Fresnel reflection (<3.5%), a good visibility in the resulting fringe pattern.

**Figure 2.**
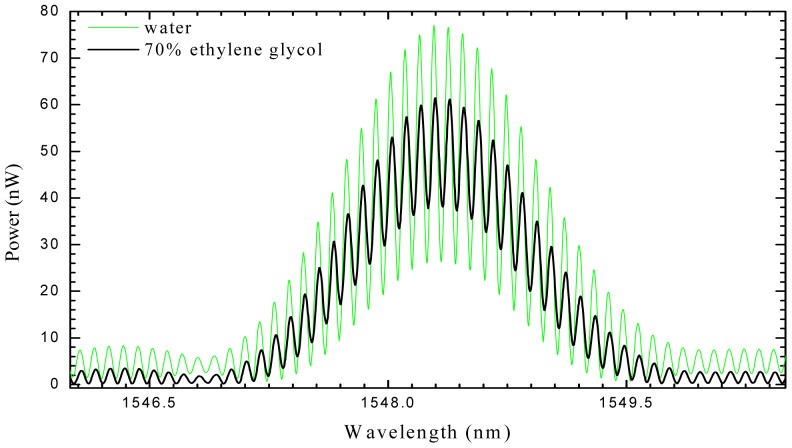
Channeled spectra of the Fabry-Perot cavity, showing the changes in fringe visibility when going from water to a solution of 70% ethylene glycol.

The refractive index information is derived from the interferometer fringe visibility, directly when the readout instrument is an optical spectrum analyzer (as can be seen in Figure 2), or using the amplitude of the carrier when a pseudo-heterodyne technique is applied to interrogate the interferometer. The sensing head was exposed to solutions with different refractive index in the range between 1.333–1.405. The resolution of 10^−3^ RIU was determined. Gouveia *et al.* [12] presented a similar sensing device for temperature and refractive index measurement, based in the same principle but using a Panda Hi-Bi fiber. Due to the fiber birefringence, two FBGs are generated resulting in two closely spaced interference patterns each with different sensitivities to refractive index and temperature. The different sensitivities allow the possibility of multiparameter discrimination. The refractive index was read from the visibility of the fringes patterns and the temperature can be measured through the wavelength shift of the gratings. The sensor was characterized in the range 1.335–1.375 and the estimated resolution was 10^−3^ RIU.

A LPG/FBG hybrid configuration was also presented by Gouveia *et al.* [13] for simultaneous measurement of refractive index and temperature. The sensing head consisted of three gratings, one LPG followed by two FBGs. The spectral position of each grating was chosen in order to have one reflection peak on each side of the LPG resonance. In this scheme the resonant peak of the LPG shifts in wavelength in accordance with the variations of the refractive index of the surrounding medium. This perturbation thus changes the intensity of light reflected by the two FBGs. The refractive index was measured by the ratio between the intensities reflected by the two FBGs. This ratio is proportional to the wavelength shift, and thus to the external refractive index, and it is independent of any other optical power fluctuations. Temperature measurement was obtained by monitoring the wavelength shift of either one of the FBGs. The sensing head was subjected to a variation of refractive index between 1.335–1.375. The estimated resolution was 2 × 10^−5^ RIU.

## 3. Long Period Gratings

Long-period gratings (LPGs) can be considered a special class of fiber Bragg gratings in which the period of the index modulation is such that it satisfies a phase matching condition between the fundamental core mode and a forward propagating cladding mode of an optical fiber.

For a LPG, a periodic modulation of the refractive index in the fiber core has, typically, a period in the range from 100 μm to 1,000 μm and a length of a few centimeters, which is induced in the optical fiber using different, but common techniques: UV laser irradiation, electric-arc discharge, periodic etching, CO_2_ laser irradiation and mechanical processes.

Thus, LPGs are intrinsically sensitive to external refractive index displaying changes in the position of the resonance wavelength. The first long period grating inscribed successfully in an optical fiber was described in 1996 by Vengsarkar *et al.* [4] for band-rejection filters, and in the same year Bhatia *et al.* [14] presented the first application of long period gratings for refractive index sensing, reporting an average resolution of 7.69 × 10^−5^ RIU in the 1.404–1.452 refractive index range. After that, many refractive index sensors using a single long-period grating have been reported along the years, using the refractometric ability to measure parameters such as the concentration of chloroform, sodium chloride or ethylene glycol [15–19].

In INESC Porto, in 2006 Rego *et al.* [20] presented the first refractive index sensor using single LPG written by electric-arc discharges in SMF 28 fiber and pure-silica-core fibers. The authors showed that LPGs written in pure-silica-core fibers possess higher sensitivity compared with those induced in standard fibers, showing sensitivities up to 40 nm/RIU. In the same year Falate *et al.* [21] presented a refractive index sensor based on a phase-shifted long-period fiber grating written by electric-arc discharges. With standard tracking of the wavelength peak shift, the resolution reported was ∼1 × 10^−3^ RIU. The main advantage of using phase-shifted LPG sensors instead of conventional LPGs is the positive peak presence in the transmission spectrum. Such a positive peak allows a high power level to be available when the wavelength measurement is performed with improvement in the signal-to-noise ratio of the refractive index measurement system. In fact, using a single laser line at a fixed wavelength in one of the slopes of the LPG transmission, and monitoring the transmitted power modulation induced by the resonance shift, a sensitivity of 146 μW/RIU was estimated. If a resolution of 10 nW is considered for the detection system this can translate into a resolution of 6.8 × 10^−5^ RIU. Caldas *et al.* [22] in 2010 presented a refractive index sensor using a single arc-induced LPG in a B/Ge co-doped fiber with a dual resonance. Measurements with LPGs for external refractive index in the range from 1.33 to 1.43 and two different situations were considered: one when the grating was straight and the other when it had a fixed curvature. On account of the asymmetric nature of electric-arc based LPGs, it was expected that application of curvature to these devices would cause a redistribution of the evanescent field impacting the sensitivity to external refractive index. In fact, the results showed that the sensitivity to external refractive index could be tuned by introducing a controlled curvature in the LPG. In the same year, Abe *et al.* [23] presented a sensor based on a refractive index change for analysis of the quality of food oils (having refractive index higher than that of the fiber cladding) using a LPGs coated with a Langmuir–Blodgett (LB) film. In particular, coatings were designed to optimize the response of the LPG when the external refractive index had a value higher than that of the cladding. In this range of refractive index, standard LPGs are characterized by a much lower sensitivity. However, by coating the device with films having adequate thickness and refractive index, the effective refractive index of the cladding modes could be adjusted in order to improve the device sensitivity. The results obtained showed that the deposition of the thin films effectively improved the response of the structure to changes in the refractive index of the external media.

**Figure 3.**
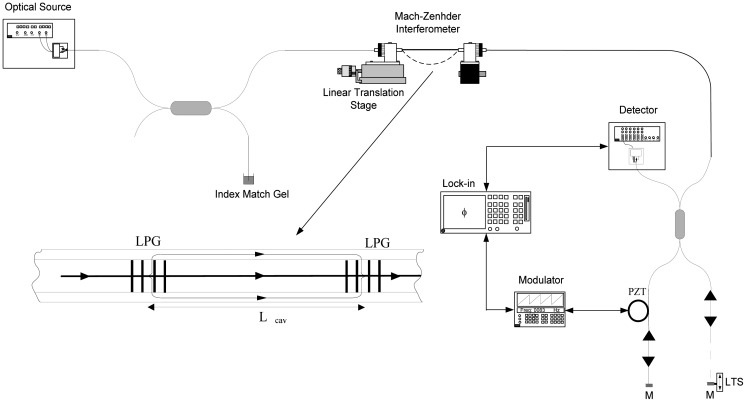
Interrogation system for LPG based Mach-Zehnder sensing structure. Adapted from [30].

Another possibility to increase the sensitivity to the external medium is to build LPG-assisted fiber interferometers with Michelson or Mach-Zehnder layouts [24–28]. In 2008 Caldas *et al.* [29] presented a long-period-grating-based fiber optic Michelson modal interferometer with coherence addressing and heterodyne interrogation as a sensing structure for measuring environmental refractive index. With such approach, the spectral shift of the interferometric fringes is readout as a phase shift of a carrier signal, allowing for increased resolution. Typically, phase readings allow for an increase of resolution of about an order of magnitude as compared to the standard spectral reading of the same sensor. In this particular case, resolution values of 9.0 × 10^−5^ RIU and 4.6 × 10^−5^ RIU were obtained for the standard and etched fibers, respectively. These figures, however, were limited by the relatively high phase noise of the particular interferometer used due to practical limitations in system stabilization. However, proper stabilization of the interferometer to values reported in the literature could improve these results by one or two orders of magnitude. In 2009, Caldas *et al.* [30] presented a modal interferometer based on LPGs in a Mach–Zehnder configuration with pseudo-heterodyne processing for refractive index measurements. In this work fiber sensing interferometers were illuminated by a broadband source. The spectrally modulated signals were then carried through a receiving interferometer (see Figure 3). The receiving interferometer, in a Michelson configuration had, in one of its arms, the possibility to tune the path length by adjusting the distance between a mirror and a GRIN lens. This way, the overall path imbalance of the receiving and sensing interferometers could be tuned to be within the optical source coherence length. In these conditions, by modulating the phase of the receiving interferometer, using a ring PZT with fiber wrapped around, it was possible to generate a carrier signal whose phase contained the information on the measurand induced spectral shift in the sensing interferometer. This phase tracking technique allows following the spectral shifts with very high resolution.

**Figure 4.**
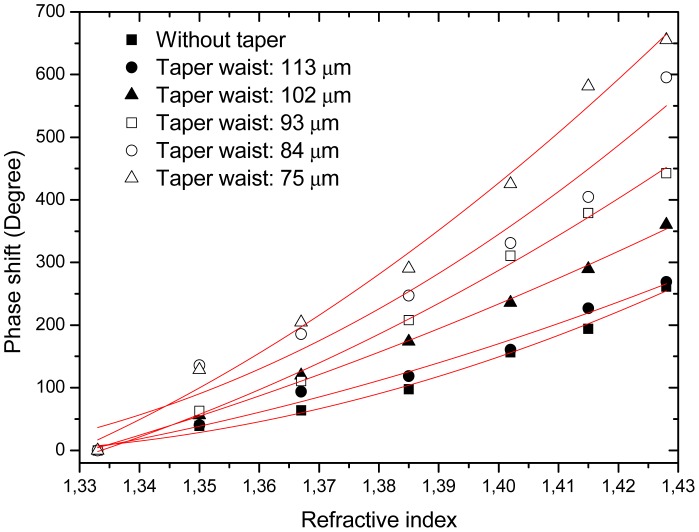
Sensing system phase changes due to refractive index variations for a tapered LPG-based Mach-Zehnder interferometer with different taper waist diameters.

A study was carried out showing that the system sensitivity could be tuned by carefully controlling the curvature of the fiber in the interferometric path. Tapering of the fiber section between the two LPGs was also shown to allow some tuning of the device sensitivity. As it can be seen from the results in Figure 4, the refractive index sensitivity of LPG Mach-Zehnder using fibers with different tapering diameters increases as the taper waist diameter is reduced down to 75 μm.

## 4. Multimodal Interference

Multimode interference (MMI) in optical fiber devices is usually obtained by splicing a multimode fiber (MMF) section between two single mode fibers (SMF), thus forming a SMF-MMF-SMF (SMS) fiber configuration. The SMS fiber concept relies on the fact that when the light field coming from the input SMF enters the MMF, will excite several modes, propagating along the MMF section, thus causing interference between them. This means that the optical power coupled to the output SMF will depend on the amplitudes and relative phases of the various modes at the exit end of the MMF. Also, the SMS fiber structure can act as a bandpass filter or edge filter depending on the length of the MMF used.

MMI fiber devices are very attractive due to their high potential for refractive index sensing. In 2006, Wang *et al.* [31] presented the first numerical simulation for an optical fiber refractometer based on MMI—the sensing structure was a 50 μm-diameter MMF core section spliced between two SMFs where the surrounding liquid worked as the cladding medium. The refractive index resolution was estimated to be 5.4 × 10^−5^ RIU for a refractive index range from 1.33 to 1.45. Recently, Wu *et al.* [32] investigated the influence of etched-MMF core diameters and lengths on the sensitivity of an SMS fiber based-refractometer. They have shown that the MMF diameter had significant influence on the refractive index sensitivity but no dependence on the fiber length. For the sensing structure with the lowest MMF core diameter (80 μm), a maximum sensitivity of 1815 nm/RIU was attained in the refractive index range 1.342–1.437. In another perspective, a simple tapering technique was proposed and experimentally demonstrated in order to increase the sensitivity of an SMS fiber structure to refractive index [33]. The sensing device was based on a tapered-MMF section sandwiched between two SMFs and relied on the evanescent field interaction between the tapered fiber and the surrounding medium. A maximum sensitivity of 1913 nm/RIU was achieved with a 30 μm-MMF taper waist diameter.

**Figure 5.**
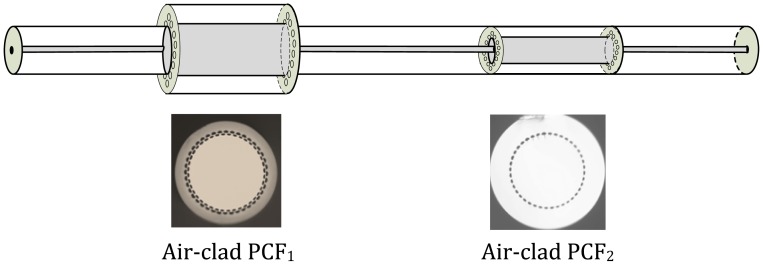
Detail of the PCF-based sensing structure (the cross section of the PCF fibers is also shown). Adapted from [34].

In this line of research, at INESC Porto, in 2011 Silva *et al.* reported the first MMI fiber-based refractometer which consisted in a SMS fiber structure relying on a large-core, air-clad photonic crystal fiber (PCF) [34]. This configuration is shown in Figure 5.

**Figure 6.**
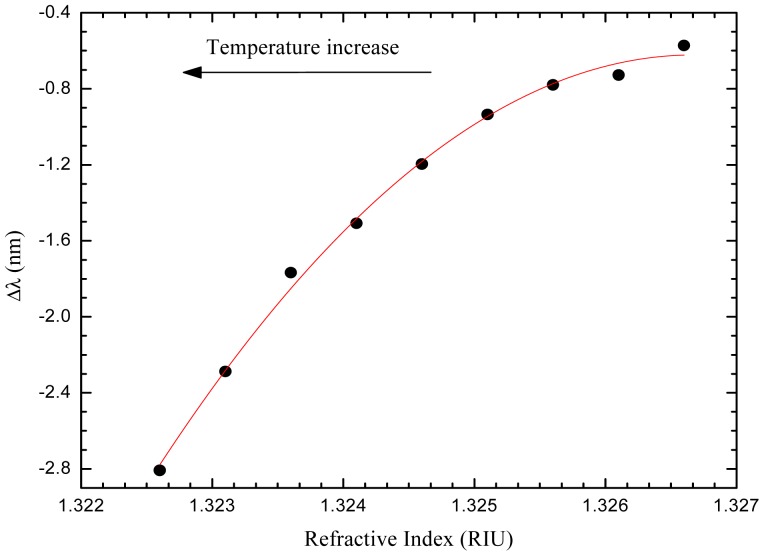
Refractive index response of the sensing head to temperature variations of water.

Two distinct large-core air-clad PCF geometries were implemented: one had a ring of air holes in contact with the external medium, so it was used for refractive index measurement (air-clad PCF1), and the other was used for temperature compensation (air-clad PCF2), since the air holes were obstructed in the splice zone. This sensing head allowed measuring refractive index changes induced by temperature variations in water by tracking the wavelength shift of the fringe pattern. A maximum sensitivity of 800 nm/RIU was attained in the refractive index range 1.3216–1.3246 and a resolution of 3.4 × 10^−5^ RIU was achieved (results shown in Figure 6).

**Figure 7.**
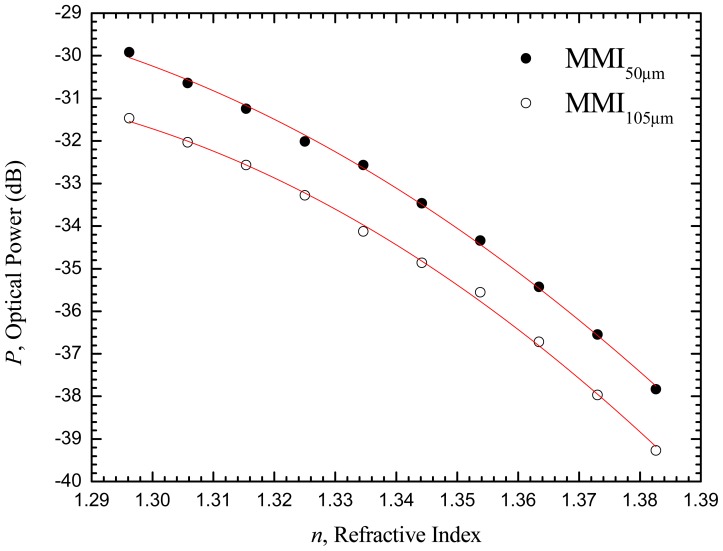
Variation of optical power, *P*, with refractive index, for MMI-based fiber tips with 50 and 105 μm MMF core diameters, respectively.

Later, a review of MMI fiber-based refractometers was presented and a simple low-cost interrogation technique relying on FBGs was proposed [35]. Recently, a reflective MMI fiber-based sensor was developed for the measurement of refractive index variations by means of intensity variation based on the fiber tip-interaction concept [36]. Resolutions of 2.2 ཌ 10^−4^ RIU and 3.8 ཌ 10^−4^ RIU were achieved for MMF tips with core diameters of 50 μm and 105 μm, respectively. From the results obtained (displayed in Figure 7), a maximum sensitivity of −110 dB/RIU was obtained for both fiber sensors in the refractive index range from 1.33 to 1.38.

## 5. Micro-Interferometers

Ran *et al.* [6] proposed a Fabry-Perot (FP) optical fiber sensor for refractive index measurements. The sensor head was fabricated by producing a circular hole with ∼23 μm of depth and ∼56 μm of diameter at the center of the cross section of a SMF by using a 157 nm laser micromachining system. This micromachined fiber was then spliced to another fiber to enclose the air cavity. The proposed sensor has three FP cavities, the first one is due to the reflection glass/air, the second one is the result of the air/glass reflections and finally the third one is due to the reflections glass/surrounding medium. The external refractive index was determined according to the maximum fringe contrast of the interference fringes in the reflective spectrum of the sensor, resulting a resolution of 4 × 10^−5^ RIU.

Rao *et al.* [37] developed a fiber optic refractive index sensor based on an intrinsic FP cavity. In this case the intrinsic FP cavity is created by a section of an endlessly photonic crystal fiber (PCF) spliced in each end to a SMF. The sensor was tested from 20 to 100 °C showing a temperature sensitivity of 4.16 nm/°C (fringe spectral shift) and a refractive index sensitivity of 4.59/RIU (fringe visibility change).

Deng *et al.* [38] constructed a novel refractive index sensor based on an optic fiber FP interferometer by splicing a section of a hollow core fiber, of pure silica, between a SMF and a PCF. The holes in the PCF allow liquids and gases to enter and leave the air cavity formed by the hollow core fiber. There are Fresnel reflections between the SMF and the hollow core fiber, between the hollow core fiber and the PCF, and finally between the PCF and the external medium. The authors tested the sensor as a refractometer in air with different pressures resulting in refractive index changes between 1.000266 and 1.000419, which resulted in a sensitivity of 805.1 μm/RIU.

Gong *et al.* [5] developed a fiber optic FP refractive index sensor for liquids able to also measure temperature. The sensor was fabricated by etching a GI-MMF with HF 40% during 4 minutes creating a concavity in the fiber end and then splicing to a SMF forming an inner air gap. The focusing effect of the GI-MMF was studied by using sensor with different lengths. By the fringe contrast and wavelength shift the authors showed that the sensor developed has a sensitivity of ∼45.1 dB/RIU and 11.5 pm/°C to refractive index and temperature, respectively.

In INESC Porto, a micrometric Fabry-Perot refractometer based on an end-of-fiber polymer tip was reported in 2009 [39]. The sensitivity is approximately 4650 deg/RIU. The limitation of this sensing head is the temperature–cross sensitivity due to the high thermal expansion of the polymer.

Frazão *et al.* [40] also proposed a refractive index sensor for liquids by splicing SMF to each end of a fragment of a suspended core fiber with three hollows and with a length of ∼210 μm. The suspended core between the two SMF creates a physical cavity that works as reference because this cavity is isolated from the refractive index changes. The sensor presents three FP cavities, the first one is between the SMF and the suspended core fiber, the second one is between the suspended core and the second SMF and finally the third FP cavity is between the second SMF and the surrounding medium. This last cavity is the one used for the refractive index measurements. The sensor was tested by measuring the fringe visibility and through the fast Fourier transform of the fringe pattern. These parameters showed distinct behaviors. A sensitivity of the visibility was estimated in −2.03 RIU^−1^ with a resolution of 7 × 10^−4^ RIU was obtained by monitoring the fringe visibility. On the other hand, when looking at the ratio of the FFT peaks (corresponding to different frequencies of the fringe pattern) a sensitivity of the normalized amplitude was estimated in −11.27 RIU^−1^ and a resolution of 2 × 10^−4^ RIU, were obtained instead.

**Figure 8.**
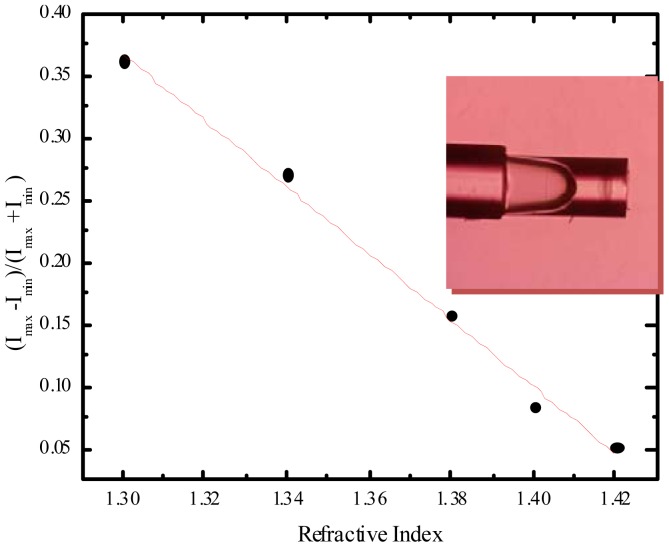
Sensitivity of the sensing head (inset: microscopic image of the sensing head).

Recently INESC Porto developed a sensor by etching GI-MMF during 10 minutes in 48% HF resulting in a concavity of 272 μm. Then this etched GI-MMF was spliced to SMF giving the first FP cavity, through the Fresnel reflections of the interface air/glass of the SMF and the interface air/glass of the GI-MMF. To obtain the second cavity the GI-MMF was cleaved approximately 2 mm of the splice between the two fibers. This cavity is due to the Fresnel reflections glass/air and it is sensible to the surrounding medium. The geometry and the linear response of the sensing head are shown in Figure 8. The sensitivity of the sensor was tested by analyzing the changes in the visibility of the exterior cavity that is directly dependent on the refractive index. The interior cavity may be used as a temperature sensor. The sensor proposed has a sensitivity of −2.67 dB/RIU.

## 6. Sensors Based on Cladding Mode Resonance

In this section, it is presented sensors based in cladding mode resonances that include tapers and capillary tubes. One of the first refractometers with a tapered optical fiber was presented by Kumar in 1984 [2]. It used a multimode plastic-clad silica core fiber and in a small section the cladding was removed and tapered by electrical spark-heating. The optical power in the output was dependent on the refractive index around the taper. The normalized power obtained for different values of refractive index was compared with theoretical results showing a very good agreement.

In 2005 Monzon-Hernandez *et al.* [41] presented a cladded multimode tapered fiber tip as a refractometer. The taper was produced by stretching the graded index fiber while it is heated with an oscillating flame torch. When ready it is cleaved in the middle and two sensing tips are created with less than 80 μm diameter. The end face is coated with gold to produce a mirror. The working principle is based on the radiation modes guided by the cladding which is influenced by the refractive index of the surrounding medium. The results indicate that is possible to measure refractive index changes of 3 × 10^−5^ with a tip of 60 μm diameter.

One year later Kieu *et al.* [42] published a paper where a sensor for a refractive index measurement with a biconical fiber taper structure is presented. The taper was produced by heating and stretching a single mode fiber (SMF28) with 16 mm length. The transmitted spectrum is nearly sinusoidal and shifted in wavelength accordingly to the refractive index medium surrounding the sensing region. The sensitivity achieved was around 705 nm/RIU at ྔ∼1550 nm in a range of refractive index from 1.333 to 1.350.

**Figure 9.**
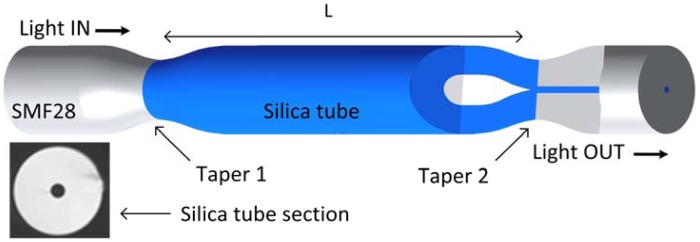
Schematic diagram of the measurement setup with a section of silica tube spliced and tapered between two single mode fibers. Adapted from [43].

Recently, two multimode interferometers based-fiber optic sensors were studied at INESC Porto by Coelho *et al.* to measure refractive index variations of the surrounding liquid. In one of them, presented in 2011, a silica tube section as a sensing head was used [43]. The silica tube was fusion spliced and tapered to single mode fibers forcing the light to be guided in the silica tube (Figure 9).

**Figure 10.**
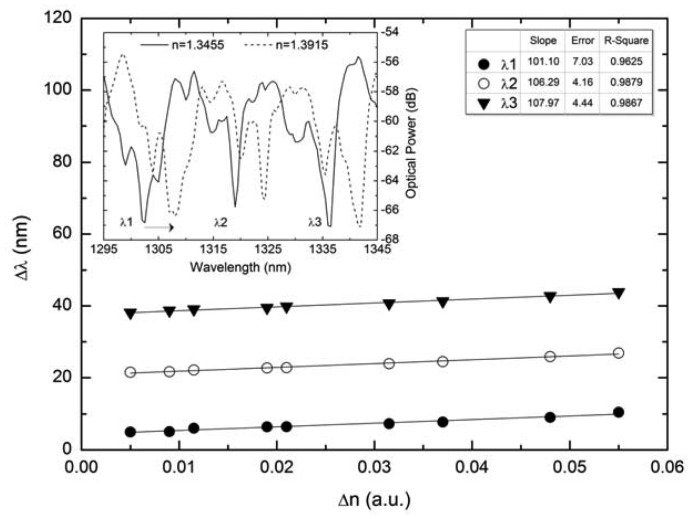
Wavelength shift of different peak as a function of the surrounding refractive index. Inset: Typical spectral output of the silica tube sensors.

The result is a multimode interference in the silica tube limited by the air in the hole and the surrounding medium both with lower refractive index. The transmitted spectra show some resonances at a certain wavelength similar to reject bands, which means that residual light is coupled to the output SMF at these wavelengths. When the external refractive index increases a red shift of these resonances is observed. A typical response of such devices to refractive index changes is shown in Figure 10, where the spectral shift of different peaks (see inset in the same figure) is given as function of surrounding refractive index. Two lengths of silica tube were compared, 8.5 cm and 17 cm for a refractive index range from 1.341 to 1.394. The sensitivity to refractive index variations obtained with this configuration (lower length) was 108 nm/RIU at ྔ∼1335 nm, while for the higher length a value of 112.1 nm/RIU at 1280 nm wavelength was registered. For the second configuration, presented in 2012 [44], a large-core photonic crystal fiber-based sensing structure was considered, being the light guided in the outer cladding. Two single mode fibers were spliced to a PCF section of 5 cm long and the SMF core was aligned with the outer cladding PCF without closing the holes. The result is once again a series of transmitted resonances that could be used as sensing feature for refractive index variations. The sensitivity achieved was 322 nm/RIU with a resolution of 7.2 × 10^−4^ RIU. Both multimode interferometers presented low temperature dependence due to use of the pure silica PCF fibers as sensing elements.

In another development, a single-mode nonadiabatic tapered optical fiber sensor inserted into a fiber loop mirror was studied [45]. Its main feature is the possibility to tune the refractive index measurement sensitivity by control of the polarization inside the loop. It was demonstrated that sensitivity could be controlled in the range from 876 nm/RIU to 1233 nm/RIU. Therefore, such configuration would allow, considering a reading system with a 1 pm resolution, a refractive index resolution better than 10^−6^ RIU.

## 7. Plasmonic Structures

Surface plasmon resonance (SPR) has become the standard technique used for a huge variety of chemical sensors or biosensors, mainly in the so-called Kretchmann configuration [46]. Plasmon excitation strongly depends on the refractive index of the surrounding medium, and, in that sense, all these sensors can be considered to be refractometers. Considering the SPR phenomenon in planar waveguides, the polarization of the incident light plays a critical role because only TM-polarized incident light will excite plasmons. The plasmon excitation produces a minimum in the spectral transmittance for a certain spectral region and refractive index values.

Devices exploiting this sensitivity of the surface plasmons are widely used for the implementation of chemical and biosensors [47]. The relative ease of manipulating surface plasmons on the surface and their intrinsic two-dimensional nature opens an opportunity for their application to photonics and optoelectronics for scaling down optical and electronic devices to micrometric dimensions. The use of optical fibers for SPR sensing was first proposed by Jorgenson and Yee [4] in 1993. In this work the sensing element was built by removing a section of the fiber cladding and depositing an SPR active thin metal layer symmetrically around the fiber core. This work was based on a resonant wavelength interrogation, and this sensing structure was the beginning of a new concept of SPR miniaturized sensors. In particular, tapered optical fibers, D-fibers or even polished optical fibers have constituted useful alternatives to the classical configurations in these application fields [48,49].

**Figure 11.**
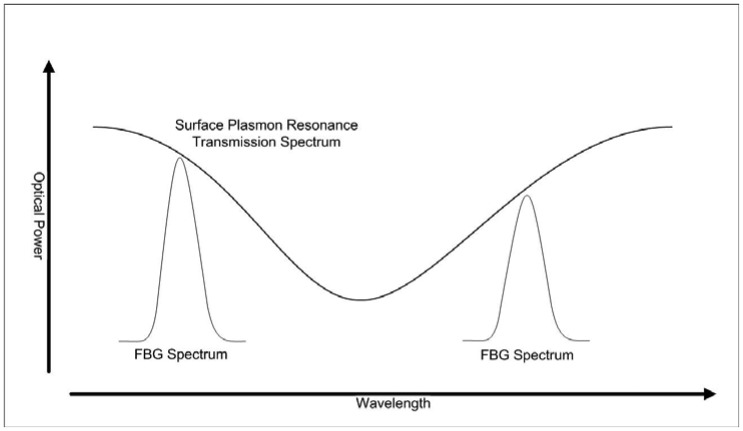
Schematic view of the interrogation system based on FBGs.

**Figure 12.**
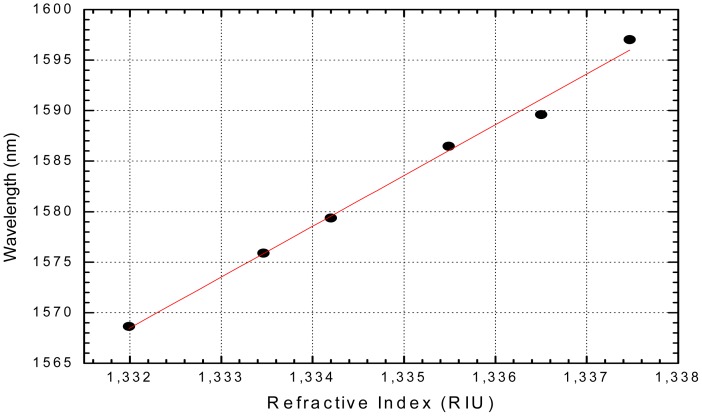
Peak wavelength response of the SPR sensor to different refractive index solutions.

In Portugal, the first work in surface plasmon resonance for fiber sensing was published by Díaz-Herrera and Viegas in 2010 [50]. In this work a new configuration of a refractometric sensor for aqueous solutions based on the combination of surface plasmon resonance (SPR) with fiber Bragg gratings (FBG) is presented. Two FBGs are selected having reflection maxima in each side of the plasmon resonance peak. A schematic view of the spectral arrangement of the gratings can be seen in Figure 11. These FBGs enable a different processing scheme for the information provided by the SPR transducer. This improved interrogation method increases the sensitivity and resolution of the sensor when compared with those obtained with the usual method of tracking the spectral transmittance minimum and makes the system performance independent of optical source power fluctuations. The experimental results obtained with a double layer uniform-waist tapered fiber show the feasibility of this approach and its applicability in SPR-based biosensors that must face very exigent measuring conditions. The resolution achieved in this configuration can be as good as 2 × 10^−5^ RIU, which is one order of magnitude better than that obtained with the typical scheme of analysis for these SPR transducers (peak wavelength response shown in Figure 12).

Taking advantage of all the devices for optical communications available at the spectral window around 1.5 μm, Viegas *et al.* [51] published in 2010 a new SPR optical fiber sensor operating at 1.5 μm and based on doubly deposited tapered fiber structures, well known to exhibit small or zero sensitivity to polarization. It was shown this configuration allows a substantially higher sensitivity compared to the ones obtained with the same elements in the 800 nm region (typically, ∼5000 nm/RIU at 1500 nm *vs.* ∼3000 nm/RIU at 800 nm). So far, the highest sensitivity reported of an optical fiber based SPR sensor was associated to a small solid core Bragg fiber resulting in a value of ∼12,000 nm/RIU [52].

## 4. Conclusions

A review was presented on the main optical fiber based sensing configurations used to measure refractive index, with focus on those derived from the R&D activity of INESC Porto. Bragg grating technology in this sensing field has some advantages mainly associated with its intrinsic wavelength multiplexing ability. Long period gratings present high sensitivity to refractive index measurement but cross sensitivity to others parameters can be a limitation. On the other hand, new designs of microstrutured fibers provide new sensing opportunities. Multimode interference sensing presents also interesting solutions because they can be easily fabricated and applied in different situations. This type of sensor has been mainly reported in the last two years with novel configurations. However, multiplexing multimode interference sensors for refractive index measurement is still a challenge. Micro-interferometers based on microstrutured fibers represent a new area recently explored by the research community. Their main advantages are the small dimensions and its high sensitivity to refractive index, although the multiplexing ability needs yet to be confirmed. Structures based on cladding etching are easily fabricated but present some fragility after the fabrication, requiring careful packaging. The last addressed technology in this review is based on surface plasmonic resonance and is highly promising in face of the extreme sensitivities it allows for refractive index sensing. Table 1 summarizes the main features of the refractometric configurations developed at INESC Porto to date.

Overall the configurations presented here constitute a library of refractometric optical fiber platforms covering a wide range of sensitivities and possessing different physical and geometrical characteristics enabling a diversity of applications. In particular, refractometric based fiber optic devices are highly attractive for chemical and biological label-free sensor technology and some of the configurations reported here are being explored in this context in applications that range from environmental monitoring to biotechnology, energy and food industries.

## Figures and Tables

**Table 1.** Summary of most relevant optical fiber based refractometers developed at INESC Porto.

**Configuration**	**Measurement Method**	**Year of Publication**	**Refractive Index Range**	**Sensitivity**	**ResolutionRIU**	**Reference**
**Etched FBG**	Spectral shift	2004	1.330–1.341	727 nm/RIU	10^−4^	[9]
**FBG in etched H fiber**	Spectral shift	2009	1.333–1.390	3.5 nm/RIU	5 × 10^−3^	[10]
**FBG/Fabry-Perot**	Carrier visibility	2008	1.333–1.405	0.6 mV/RIU	10^−3^	[11]
**FBG/Fabry-Perot Hi-Bi Fiber**	Fringe Visibility	2012	1.335–1.375	1%/0.01 RIU	10^−3^	[12]
**Hibrid FBG-LPG**	Amplitude modulation	2009	1.335–1.375	4%/0.001 RIU	2 × 10^−5^	[13]
**LPG in Pure silica fiber**	Spectral shift	2006	1.3211–1.3271	40 nm/RIU	-	[20]
**Pi-Shifted LPG**	Amplitude modulation	2006	1.332–1.342	146 μW/RIU	6.8 × 10^−5^	[21]
**LPG based interferometer**	carrier phase tracking	2008	1.40–1.43	26,700 deg/RIU	4.6 × 10^−5^	[29]
**Air clad PCF SMS structure**	Spectral shift	2011	1.3216–1.3246	800 nm/RIU	3.4 × 10^−5^	[34]
**50 μm MMI fiber tip**	Amplitude modulation	2012	1.33–1.38	110 dB/RIU	2.2 × 10^−4^	[36]
**Polymer tip Fabry Perot**	carrier phase tracking	2009	1.333–1.450	4650°/RIU	7.5 × 10^−4^	[39]
**Suspended Core fiber Fabry Perot**	FFT analysis	2009	1.33–1.424	−11.27/RIU	2 × 10^−4^	[40]
**Etched GI fiber Fabry Perot**	Fringe visibility		1.30–1.42	−2.67 dB/RIU	-	unpublished
**Silica tube Fabry Perot**	Spectral shift	2011	1.341–1.394	112.1 nm/RIU	-	[43]
**PCF fiber cladding MMI**	Spectral shift	2012	1.331–1.373	322 nm/RIU	7.2 × 10^−4^	[44]
**Non adiabatic taper in fiber loop mirror**	Spectral shift	2011	1.338–1.352	1233 nm/RIU	1 × 10^−6^	[45]
**SPR-FBG**	Amplitude modulation	2010	1.333–1.340	20%/0.01 RIU	2 × 10^−5^	[50]
**SPR-taper**	Spectral shift	2010	1.332–1.338	5000 nm/RIU	-	[51]

## References

[1] Cooper P.R. (1983). Refractive-index measurements of liquids used in conjunction with optical fibers. Appl. Opt..

[2] Kumar A., Subrahmanyam T.V.B., Sharma A.D., Thyagarajan K., Pal B.P., Goyal I.C. (1984). Novel refractometer using a tapered optical fiber. Electron. Lett..

[3] Asseh A., Sandgren S., Ahlfeldt H., Sahlgren B., Stubbe R., Edwall G. (1998). Fiber optical bragg grating refractometer. Fiber Integr. Opt..

[4] Vengsarkar A.M., Lemaire P.J., Judkins J.B., Bhatia V., Erdogan T., Sipe J.E. (1996). Long-period fiber gratings as band-rejection filters. J. Light. Technol..

[5] Gong Y., Guo Y., Rao Y.-J., Zhao T., Wu Y. (2010). Fiber-optic fabry-perot sensor based on periodic focusing effect of graded-index multimode fibers. IEEE Photonics Technol. Lett..

[6] Ran Z.L., Rao Y.J., Liu W.J., Liao X., Chiang K.S. (2008). Laser-micromachined Fabry-Perot optical fiber tip sensor for high-resolution temperature-independent measurement of refractive index. Opt. Express.

[7] Jorgenson R., Yee S. (1993). Fiber-optic chemical sensor based on surface plasmon resonance. Sens. Actuators B Chem..

[8] Frazão O., Ferreira L.A., Araújo F.M., Santos J.L. (2005). Applications of fibre optic grating technology to multi-parameter measurement. Fiber Integr. Opt..

[9] Pereira D.A., Frazão O., Santos J.L. (2004). Fibre Bragg grating sensing system for simultaneous measurement of salinity and temperature. Opt. Eng..

[10] Frazão O., Martynkien T., Baptista J.M., Santos J.L., Urbanczyk W., Wojcik J. (2009). Optical refractometer based on a birefringent Bragg grating written in an H-shape fiber. Opt. Lett..

[11] Silva S.F.O., Frazão O., Caldas P., Santos J.L., Araújo F.M., Ferreira L.A. (2008). Optical fibre refractometer based on a fabry-pérot interferometer. Opt. Eng..

[12] Gouveia C., Jorge P.A.S., Baptista J.M., Frazão O. (2012). Fabry-Pérot cavity based on a high-birefringent fiber Bragg grating for refractive index and temperature measurement. IEEE Sens. J..

[13] Jesus C., Caldas P., Frazão O., Jorge P.A.S., Baptista J.M., Santos J.L. (2009). Simultaneous measurement of refractive index an temperature using a hybrid FBG/LPG configuration. Fiber Integr. Opt..

[14] Bhatia V., Vengsarkar A.M. (1996). Optical Fiber Long-Period Grating Sensors. Opt. Lett..

[15] Khaliq S., James S.W., Tatam R.P. (2001). Fiber-optic liquid-level sensor using a long-period grating. Opt. Lett..

[16] Falciai R., Mignani A.G., Vannini A. (2001). Long Period gratings as solution concentration sensors. Sens. Actuators B Chem..

[17] Pilla P., Iadicieco A., Contessa L., Campopiano S., Cutolo A., Giordano M., Guerra G., Cusano A. (2005). Optical chemo-sensor based on long period gratings coated with delta form syndiotactic polystyrene. IEEE Photonics Technol. Lett..

[18] Chen X., Zhou K., Zhang L., Bennion I. (2004). Optical chemsensors utilizing long-period fiber gratings UV-inscribed in D-fiber with enhanced sensitivity through cladding etching. IEEE Photonics Technol. Lett..

[19] Rees N.D., James S.W., Tatam R.P., Ashwell G.J. (2002). Optical fiber long-period gratings with langmuir-blodgett thin-film overlays. Opt. Lett..

[20] Rego G.M., Santos J.L., Salgado H.M. (2006). Refractive index measurement with long-period gratings arc-induced in pure-silica-core fibres. Opt. Commun..

[21] Falate R., Frazao O., Rego G., Fabris J.L., Santos J.L. (2006). Refractometric Sensor based on a phase-shifted long-period fiber grating. Appl. Opt..

[22] Caldas P., Rego G., Ivanov O.V., Santos J.L. (2010). Characterization of the Response of a Dual Resonance of an Arc-Induced Long-Period Grating to Various Physical Parameters. Appl. Opt..

[23] Abe I., Oliveira J., Simoes E., Caldas P., Frazao O. (2010). Monitoring the quality of frying oils using a nanolayer coated optical fiber refractometer. Talanta.

[24] Dianov E.M., Vasiliev S.A., Kurkov A.S., Medvedkov O.I., Protopopov V.N. In-Fiber Mach-Zehnder Interferometer Based on a Pair of Long-Period Gratings.

[25] Allsop T., Reeves R., Webb D.J., Bennion I., Neal R. (2002). A high sensitivity refractometer based upon a long period grating mach-zehnder interferometer. Rev. Sci. Instrum..

[26] Swart P.L. (2004). Long-period grating michelson refractometric sensor. Meas. Sci. Technol..

[27] Duhem O., Henninot J.F., Douay M. (2000). Study of in fiber mach-zehnder interferometer based on two spaced 3-db long period gratings surrounded by a refractive index higher than that of silica. Opt. Commun..

[28] Kim D.W., Zhang Y., Cooper K.L., Wang A.B. (2005). In-fiber reflection mode interferometer based on a long-period grating for external refractive-index measurement. Appl. Opt..

[29] Caldas P., Jorge P.A.S., Araujo F.M., Ferreira L.A., Marques M.B., Rego G., Santos J.L. (2008). Fiber modal Michelson interferometers with coherence addressing and heterodyne interrogation. Opt. Eng..

[30] Caldas P., Jorge P.A.S., Araujo F.M., Ferreira L.A., Rego G., Santos J.L. (2009). Geometrical effects on the refractive index sensitivity of Mach-Zehnder fibre modal interferometers based on long-period gratings. Meas. Sci. Technol..

[31] Wang Q., Farrell G. (2006). All-fiber multimode-interference based refractometer sensor: Proposal and design. Opt. Lett..

[32] Wu Q., Semenova Y., Wang P., Farrell G. (2011). High sensitivity SMS fiber structure based refractometer – analysis and experiment. Opt. Exp..

[33] Wang P., Brambilla G., Ding M., Semenova Y., Wu Q., Farrell G. (2011). High-sensitivity, evanescent field refractometric sensor based on a tapered, multimode fiber interference. Opt. Lett..

[34] Silva S., Santos J.L., Malcata F.X., Kobelke J., Schuster K., Frazão O. (2011). Optical refractometer based on large-core air-clad photonic crystal fibers. Opt. Lett..

[35] Frazão O., Silva S.F., Viegas J., Ferreira L.A., Araújo F.M., Santos J.L. (2011). Optical fiber refractometry based on multimode interference. Appl. Opt..

[36] Silva S.F., Frazão O., Santos J.L., Malcata F.X. (2012). A reflective optical fiber refractometer based on multimode interference. Sens. Actuators B Chem..

[37] Rao Y.-J., Deng M., Duan D.-W., Zhu T. (2008). In-line fiber Fabry-Perot refractive-index tip sensor based on endlessly photonic crystal fiber. Sens. Actuators A Phys..

[38] Deng M., Tang C.P., Zhu T., Rao Y.J., Xu L.C., Han M. (2010). Refractive index measurement using photonic crystal fiber-based Fabry-Perot interferometer. Appl. Opt..

[39] Frazão O., Caldas P., Santos J.L., Marques P.V.S., Turck C., Lougnot D.J., Soppera O. (2009). Fabry-Perot refractometer based on an end-of-fibre polymer tip. Opt. Lett..

[40] Frazao O., Baptista J.M., Santos J.L., Kobelke J., Schuster K. (2009). Refractive index tip sensor based on Fabry-Perot cavities formed by a suspended core fibre. J. Eur. Opti. Soc.-Rapid Publ..

[41] Monzon-Hernandez D., Villatoro J., Luna-Moreno D. (2005). Miniature optical fiber refractometer using cladded multimode tapered fiber tips. Sens. Actuators B Chem..

[42] Kieu K.Q., Mansuripur M. (2006). Biconical fiber taper sensors. IEEE Photonics Technol. Lett..

[43] Coelho L., Kobelke J., Schuster K., Frazão O. (2011). Optical refractometer based on multimode interference in a pure silica tube. Opt. Eng..

[44] Coelho L., Kobelke J., Schuster K., Frazão O. (2012). Multimode interference in outer cladding large-core, air-clad photonic crystal fiber. Microw. Opt. Technol. Lett..

[45] Zibaii M., Frazão O., Latifi H., Jorge P.A.S. (2011). Controlling the sensitivity of refractive index measurement using a tapered fiber loop mirror. IEEE Photonic Technol. Lett..

[46] Kretschmann E., Raether H. (1968). Radiative decay of non radiative surface plasmons excited by light (Surface plasma waves excitation by light and decay into photons applied to nonradiative modes). Zeitschriftfur Naturforschung Teil A.

[47] Boisdé G., Harmer A. (1996). Chemical and Biochemical Sensing with Optical Fibers and Waveguides.

[48] Villatoro J., Monzón-Hernández D., Mejía E. (2003). Fabrication and modeling of uniform-waist single-mode tapered optical fiber sensors. Appl. Opt..

[49] Slavík R., Homola J., Ctyroky J. (1999). Single-mode optical fiber surface plasmon resonance sensor. Sens. Actuators B Chem..

[50] Díaz-Herrera N., Viegas D., Jorge P., Araújo F.M., Santos J.L., Navarrete M.C., González-Cano A. (2010). Fiber-optic SPR sensor with a FBG interrogation scheme for readout enhancement. Sens. Actuators B Chem..

[51] Díaz-Herrera N., González-Cano A., Viegas D., Santos J.L., Navarrete M. (2010). Refractive index sensing of aqueous media based on plasmonic resonance in tapered optical fibres operating in the 1.5 μm region. Sens. Actuators B Chem..

[52] Hassani A., Gauvreau B., Fehri M., Kabashin A., Skorobogatiy M. (2008). Photonic crystal fiber and waveguide-based surface plasmon resonance sensors for application in the visible and near-IR. Electromagnetics.

